# Utilization of acid pre-treated coconut dregs as a substrate for production of detergent compatible lipase by *Bacillus stratosphericus*

**DOI:** 10.1186/s13568-017-0433-y

**Published:** 2017-06-23

**Authors:** Nur Bainun Mohd Zin, Busyra Mohamad Yusof, Siti Nurbaya Oslan, Helmi Wasoh, Joo Shun Tan, Arbakariya B. Ariff, Murni Halim

**Affiliations:** 1 Department of Bioprocess Technology, Faculty of Biotechnology and Biomolecular Sciences, Universiti Putra Malaysia, 43400 UPM Serdang, Selangor Malaysia; 2 Department of Biochemistry, Faculty of Biotechnology and Biomolecular Sciences, Universiti Putra Malaysia, 43400 UPM Serdang, Selangor Malaysia; 3 Bioprocessing and Biomanufacturing Research Centre, Faculty of Biotechnology and Biomolecular Sciences, Universiti Putra Malaysia, 43400 UPM Serdang, Selangor Malaysia; 4 Bioprocess Technology Division, School of Industrial Technology, Universiti Sains Malaysia, 11800 Gelugor, Penang Malaysia

**Keywords:** Lipase, *Bacillus stratosphericus*, Coconut waste, Biodetergent, Submerged fermentation

## Abstract

In recent years, many efforts have been directed to explore the methods to reduce the production costs of industrial lipase by improving the yield and the use of low-cost agricultural wastes. Coconut dregs, which is a lignocellulosic by-product from coconut oil and milk processing plants, is rich in cellulose (36%) and crude fat (9%). A newly isolated *Bacillus stratosphericus* has been demonstrated to perform cellulose hydrolysis on coconut dregs producing fermentable sugars. The highest extracellular lipase activity of 140 U/mL has been achieved in submerged fermentation with acid pre-treated coconut dregs. The lipase was found to be active over a wide range of temperatures and pHs. The activity of lipase can be generally increased by the presence of detergent ingredients such as Tween-80, cetyltrimethylammonium bromide, hydrogen peroxide and phosphate per sulphate. The great compatibility of lipase in commercial detergents has also underlined its potential as an additive ingredient in biodetergent formulations.

## Introduction

Lipases (triacylglycerol acyl hydrolase, E.C.3.1.1.3) are among the important classes of hydrolytic enzyme that can catalyse both hydrolysis and synthesis of esters (Chauhan et al. 2013). They hydrolyse triacylglycerides to fatty acids, diacylglycerol, monoacyl glycerol and glycerol at interface between oil and water (aqueous). In contrast, under non-aqueous condition, lipases will be able to catalyse reverse reactions such as esterification and trans-esterification. To date, detergent enzymes accounts for more than 37% of the world’s total enzyme production and detergent industry (Dahiya and Rathi 2014). The increasing demand for biodetergent is mostly influenced by better washing performance as compared to the synthetic detergents and the public awareness on the potential hazardous effects by chemical detergent ingredients and environmental pollution. The success of incorporating lipase in detergent formulations will promote a green technology by either partially or totally reducing the use of non eco-friendly chemical compounds.

The detergent lipases are produced by various microorganisms such as those belonged to *Staphylococcus*, *Bacillus*, *Burkholderia* and *Pseudomonas* genera (Niyonzima and More 2015). The commercially important bacterial lipases are usually extracellular enzymes and their bulk production is much easier than that of intracellular enzymes (Palaker et al. 2000). In detergent formulation, lipases are often added as either crude or partially purified form. In general, they are used as additives in detergent formulation to catalyse the removal of fat and oil stains under alkali and high temperatures (Banik and Prakash 2004). Nonetheless, to be effectively formulated as biodetergent, lipases must be able to withstand detergent ingredients including surfactants, bleaches and oxidising agents (Niyonzima and More 2015). Furthermore, they should exhibit a relatively high activity and remained their stability in alkali and high temperature conditions. Hence, continual search for novel lipases with compatibility as commercial laundry detergents is crucial to find the best functioning lipases in the washing condition without being sheared and destroyed along the washing processes.

Currently, the major bottlenecks faced by industrial enzyme producers are low yield and high production cost. For instant, media used for the production of hydrolases such as lipase generally involve expensive complex carbon and nitrogen sources that are responsible for as much as 70% raw material costs in extracellular enzyme fermentation (Jamrath et al. 2012). The exploitation of lignocelluloses materials including agroindustrial waste may hence serve as effective and cheap alternative in producing enzymes at appreciable level for commercial use. Furthermore, the use of agricultural residues for industrial purposes is more environmental friendly than other disposal methods commonly adopted nowadays. Coconut (*Cocos nucifera* L.) has been grown in more than 85 countries worldwide with Indonesia, Philippines, India, Brazil and Sri Lanka being among the top producers (Siddiq 2012). Coconut dregs is basically a residue from coconut oil and milk processing plants. This lignocelluloses biomass is abundantly available at low or without cost and commonly used as fertiliser, animal feed or simply left to decay on the fields (Sulaiman et al. 2013). Nonetheless, the utilisation of this by-product has been recently extended to more refined applications including as a nutrient rich substrate for oyster mushroom cultivation (Vetayasuporn 2007) and biodiesel production (Sulaiman et al. 2013) owing to its high coconut oil content. The solid coconut waste from coconut milk extraction process was found to have up to 24 wt% oil content (Sarina et al. 2013). In addition, the coconut waste comprises high concentration of directly fermentable carbohydrates that can be converted into free sugar by a simple chemical pre-hydrolysis during sterilisation of the medium followed by enzymatic action of the bacteria during fermentation (Ding et al. 2012).

Therefore, this paper aimed to produce lipase compatible biodetergent by utilising coconut dregs to serve as a substrate supplying carbon source as well as triglyceride inducers for efficient and cheap lipase biodetergent production.

## Materials and methods

### Isolation of lipase producers

Samples of 1 mL were taken from palm oil mill effluent (POME) sludge, hot springs and used cooking oil, whereas 1 g of sample was taken from contaminated food and mixed with 9 mL sterile distilled water. The solutions were agitated at 150 rpm under 37 °C in an incubator shaker for 30 min to enrich the microbes. The solutions were then diluted up to 10^−6^ and plated on Luria Bertani (LB) (Sigma Aldrich, United State) agar plates. The plates were incubated at 37 °C in an oven for 18 h (overnight) to obtain single colonies. The single colonies were then picked and streaked on another LB agar plates and subsequently grown for another 18 h. The steps were repeated until pure cultures of the colonies were obtained. The strains were then preserved in sterile 20% (v/v) glycerol in LB broth at −80 °C.

For screening of lipase producing bacteria, the bacteria pure cultures on the LB plates were streaked on selective plate media, which is Rhodamine B (28 g nutrient agar, 4 g sodium chloride, 10 mg rhodamine B, 10 mL olive oil in 1 L distilled water with pH 7 adjusted by the addition of sodium hydroxide prior autoclaved). The plates were incubated in an oven at 37 °C for about 1–2 days. The bacterial colonies were then observed for a pink–orange fluorescence zone hydrolysis around bacterial colony under UV 350 nm, thus indicating the positive lipase producing bacteria.

For cellulase activity test, 5 µL of overnight grown culture was spot plated on carboxymethylcellulose (CMC) agar plate (0.2% NaNO_3_, 0.1% K_2_HPO_4_, 0.05% MgSO_4_, 0.05% KCl, 0.2% CMC sodium salt, 0.02% peptone and 1.7% agar). The plates were incubated at 37 °C for 48 h. They were then flooded with Gram’s iodine for 3–5 min and observed for a zone hydrolysis around bacterial colony.

### Identification of lipase producer

Genomic DNA was extracted from the isolate using phenol–chloroform method as described by Sambrook and Russell (2001). The 16S rRNA gene was amplified with two universal eubacterial primers: fD1 and rD1. The PCR product was purified using an Agarose Gel DNA Purification Kit (Roche, Switzerland) and cloned into pTZ57R/T according to manufacturer’s structure. DNA sequencing was carried out by dideoxy chain termination method (Macrogen, Seoul, Korea) using pUC/M13 primers.

The 16S rRNA gene sequence was analysed through NCBI BLAST (http://www.ncbi.nlm.nih.gov/blast/) and EzTaxon servers (http://www.eztaxon.org/). The most similar sequences were aligned in Molecular Evolutionary Genetics Analysis (MEGA) 5.01 software and a phylogenetic tree was made by the neighbour-joining (NJ) method with 1000 bootstrap replicates in the MEGA (Tamura et al. 2011).

### Pre-treatment of coconut dregs

A sample of coconut dregs collected from local market in Serdang, Selangor, Malaysia was ground and sieved into 1 mm particle size. It was separately mixed with different concentrations of sulphuric acid (0.2, 0.6 and 1%) and sodium hydroxide (1, 3, and 5%) at 50 °C for 3 days. Pre-treatment using different concentrations of cellulase (10,000 and 30,000 U/g of cellulysin (Merck, Malaysia) was done at 40 °C and pH 4 for 3 days. One unit of cellulose was measured for decomposing activity using a filter paper substrate at 40 °C and pH 4. At the end of the hydrolysis, liquid fraction from hydrolysate was filtered to be analysed for glucose concentration by glucose assay kit (Merck, Malaysia). The residual part was dried at 80 °C until a constant weight is achieved. The dried coconut dregs was kept in a dry state and used throughout the experiments.

### Feedstock characterisation

The characterisation of untreated coconut dregs was conducted according to the acid detergent fibre, neutral detergent fibre, and acid detergent lignin (ADF–NDF–ADL) method (Goering and van Soest 1970). Hemicellulose and cellulose were calculated based on Eqs. 1 and 2.

1 $${\text{Hemicellulose (\%)}} = {\text{NDF (\%)}}-{\text{ADF (\%)}}$$

2 $${\text{Cellulose}}\,(\%) = {\text{NDF (\%)}}-{\text{Hemicellulose}}\,(\%)-{\text{Lignin}}\,(\%)$$

Meanwhile, the crude fat of untreated coconut dregs was conducted by adopting Soxhlet extraction method (Aji et al. 2015). The grounded coconut dregs were packed into the extraction chamber of the Soxhlet extractor while a solvent (*n*-hexane) was poured into the round bottom flask of the extractor. The whole set up was mounted on a heating mantle at 65 °C and allowed to reflux for about 8 h. The extract was filtered and evaporated using a rotary evaporator to isolate the free flow lipid from the solvent. The extracted oil was further evaporated in an oven at 150 °C to eliminate any moisture and residue solvent that may be present. The weight of oil produced and residue were measured to ascertain the percentage of the oil content.

The crude protein of untreated coconut dregs was conducted according to Kjeldahl method (Zainuddin et al. 2014).

On the other hand, the crude fibre of untreated coconut dregs was carried out according to that proposed by Zainuddin et al. (2014).

### Lipase production in submerged fermentation

5% overnight culture of *B. stratosphericus* PW3 (Microbial Culture Collection Unit (UNiCC), UPM: UPMC 1196; GenBank accession number: KY797998) was inoculated in a liquid medium containing 50 mL basal media (0.01% MgSO_4_·7H_2_O, 0.1% KH_2_PO_4_ and 0.5% peptone) supplemented either with 1.5% of 0.2% acid pre-treated coconut dregs, 1.5% of 5% alkali pre-treated coconut dregs, 1.5% un-treated coconut dregs, 1.5% of 0.2% acid pre-treated coconut dregs with 3.5% coconut oil or without any supplementation (control experiment). The 250 mL flasks were incubated for 32 h at pH 7, 37 °C under shaking on a rotary shaker at 200 rpm. Sampling was done at time interval and cell free supernatant obtained by centrifugation at 10,000 rpm, 10 min at 4 °C, which was considered as the crude lipase.

### Lipase assay

Lipase activity was estimated by spectrophotometric assay using *p*-nitrophenyl-laurate (pNPL) as a substrate with slightly modifications (Winkler and Stuckmann 1979). The reaction mixture comprised 0.001 mL crude lipase, 0.089 mL 0.05 M phosphate buffer (pH 7) and 0.01 mL of 0.01 M pNPL in DMSO. The mixture was incubated at 50 °C for 5 min and the reaction was stopped using 0.1 mL absolute ethanol. The absorbance was determined at 410 nm. One unit of lipase activity was calculated as µmol of *p*-nitrophenol (pNP) released per minute per millilitre of enzyme solution under standard assay conditions.

### Partial characterisation of *B. stratosphericus* lipase

#### Effects of temperature and pH on lipase activity

The optimum temperature for lipase activity of *B. stratosphericus* was determined by assaying the lipase activity at different temperatures ranging from 40 to 70 °C at pH 7 using pNPL as substrate. Relative lipase activity was measured in percentage of lipase activity of the sample with respect to the lipase activity of the control sample, which was kept at 4 °C.

Optimum pH was determined by assaying the lipase activity at different pH ranging from 4 to 10 at 50 °C in buffer solutions using pNPL as substrate. The buffer solutions used are 0.05 M sodium acetate pH 4 and 5, 0.05 M sodium phosphate pH 6, 7 and pH 8, 0.05 M glycine NaOH pH 9 and 10.

#### Effects of metal ions, inhibitor, surfactants and oxidising agents on lipase stability

The effects of various metal ions, inhibitor, surfactants and oxidising agents toward lipase activity were investigated at 1:1 (v/v) ratio with crude lipase and pre-incubated for 1 h at 50 °C. The metal ions used are 0.01 M of calcium chloride, cobalt chloride, zinc chloride, sodium chloride, potassium chloride, iron (III) chloride, manganese chloride and magnesium chloride, which contributed as Ca^2+^, Co^2+^, Zn^2+^, Na^2+^, K^+^, Fe^3+^, Mn^2+^, and Mg^2+^, respectively. For inhibitor, 0.01 M ethylenediaminetetraacetic acid (EDTA) was used. The surfactants utilised are Tween-20, Tween-40, Tween-80, Triton X-100, sodium dodecyl sulphate (SDS) and cetyltrimethylammonium bromide (CTAB). Moreover, the oxidising agents used are hydrogen peroxide (H_2_O_2_), sodium hypochlorite (NaClO), and phosphate persulphate (K_2_S_2_O_8_). The surfactants and oxidising agents were tested at different concentrations of 0.5, 1.0, 1.5, 2.0, 3.0 and 5.0%. The residual lipase activity was measured in percentage of lipase activity of the sample with respect to the lipase activity of the control sample. The control is the crude lipase incubated under similar conditions with the lipase activity taken as 100%.

#### Effects of commercial detergents on lipase stability

The stability of lipase in the presence of locally available commercial detergents was determined using 7 mg/mL (w/v) washing powders such as biodetergent 1, biodetergent 2, detergent 1, detergent 2 and detergent 3. Endogenous enzymes present in the biodetergent 1 and biodetergent 2 were first inactivated by incubating the detergent solutions at 90 °C for 1 h prior adding crude lipase from *B. stratosphericus*. The effects of commercial detergents were investigated at 1:1 (v/v) ratio with crude lipase and pre-incubated for 1 h at 50 °C. The residual lipase activity was measured in percentage of lipase activity of the sample with respect to the lipase activity of the control sample. The control is the crude lipase incubated under similar conditions with the lipase activity taken as 100%.

## Results

### Screening and identification of lipase producing bacteria

The positive lipase bacteria producers were confirmed by the production of orange–pink fluorescent colonies on Rhodamine B agar medium under UV light. Among the 22 isolates obtained from various sources including hot springs, POME sludge, spoiled foods and used cooking oil, only bacteria strains SA (isolated from hotsprings), PW3 (isolated from POME sludge) and PW2 (isolated from POME sludge) showed the highest zone formation. These three newly isolated lipase producers were further screened for bacterial cellulase activity on CMC containing plates. Based on the production of zone hydrolysis, only PW3 strain was found positive for cellulase production on CMC agar plate. The PW3 strain was then identified as *Bacillus stratosphericus* PW3 (GenBank accession number: KY797998) with 100% similarity to *Bacillus stratosphericus* 41KF2a based on 16S rRNA gene sequence analysis.

### Effects of acid pre-treatment of glucose production in coconut dregs

The proximate analysis of coconut dregs is displayed in Table 1. Coconut dreg sample was found to show 9.35% of crude fat content. From the analysis, lignocellulose of the coconut dregs was seen consisting 36.08% cellulose, 12.58% hemicellulose and 9.81% lignin.

**Table 1:** Proximate analysis of coconut dregs

Composition	Percentage, %
Crude protein	6.54
Crude fibre	27.33
Crude fat	9.35
Cellulose	36.08
Hemicellulose	12.58
Lignin	9.81

**Fig. 1:**
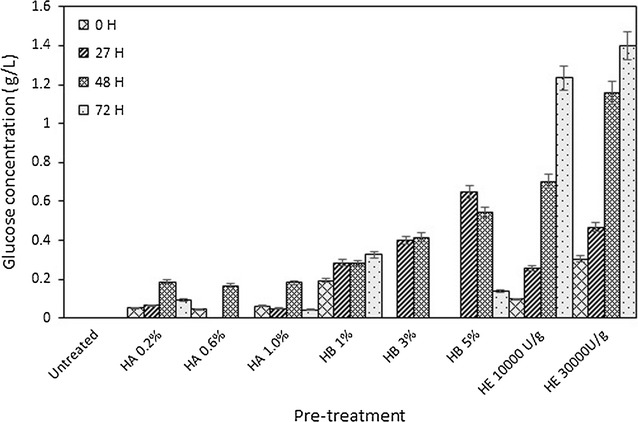
Pretreament of coconut dregs. Glucose residual was determined for coconut dregs samples that were pre-treated with different concentrations of HA: sulphuric acid, HB: sodium hydroxide and HE: cellulase for 3 days at 50 °C

Pre-treatment of the coconut dregs was conducted using different concentrations of sulphuric acid and sodium hydroxide to liberate glucose from tightly associated chain as cellulose is crystalline. Different concentrations of sulphuric acid in the range of 0.2–1% demonstrated no effect on glucose production in coconut dregs hydrolysate but treatment times substantially influenced (Fig. 1). 48 h of incubation at 50 °C was required to obtain the optimum glucose at 0.2 g/L for the tested acid concentrations. Prolonging the incubation period to 72 h has resulted in drastic reduction of glucose concentration. Unlike acid pre-treatment, alkali concentration and pre-treatment time both contributed to the glucose production. The highest glucose concentration of 0.65 g/L was achieved when coconut dregs was incubated with 5% of sodium hydroxide in 27 h. Nonetheless, glucose was dramatically decreased to 0.14 g/L at 72 h. A control experiment was performed to confirm the role of cellulase enzyme in hydrolysing cellulose to glucose from coconut dregs. The presence of commercial cellulase enzyme showed a drastic increment in glucose production from coconut dregs as 1.4 g/L glucose was achieved when 30,000 U/g cellulose present during 72 h of incubation. The ability of lipase producer *B. stratosphericus* to self-produce cellulase displayed its potential to be exploited for lipase production using lignocellulosic waste such as coconut dregs as a cheap substrate.

### Production of lipase in submerged fermentation

**Fig. 2:**
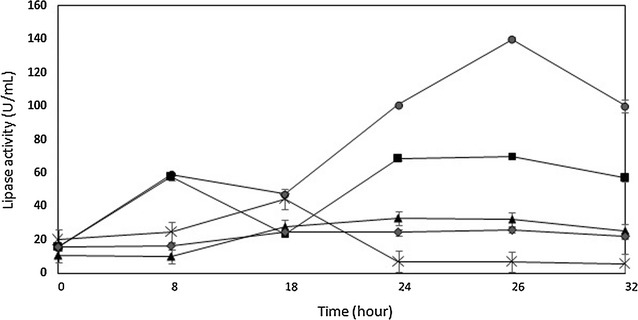
Production of *B. stratosphericus* lipase in submerged fermentation. Lipase activity was determined for submerged fermentations using (a) basal media (*diamond*); (b) basal media with acid pre-treatment coconut dregs (*circle*); (c) basal media with alkali pre-treatment coconut dregs (*square*); (d) basal media with acid pre-treatment coconut dregs and coconut oil (*triangle*); and (e) basal media with un-treated coconut dregs (χ) carbon. The fermentations were performed at 37 °C and pH 7. *Error bars* represent one standard deviation about the mean (n = 3)

Extracellular lipase production by *B. stratosphericus* using coconut dregs was performed under submerged fermentation (Fig. 2). Basal media consists of 0.01% MgSO_4_·7H_2_O, 0.1% KH_2_PO_4_ and 0.5% peptone was supplied with 1.5% of acid, alkali or untreated coconut dregs as a sole carbon source. Although glucose was found higher in hydrolysate of alkali treated as compared to acid treated, the maximum lipase activity achieved in fermentation with acid treated coconut dregs was twofold higher (140 U/mL). A low enzyme activity of 32 U/mL was quantified when the basal media in the presence of acid treated coconut dregs was supplemented with 3.5% coconut oil. Meanwhile only a maximum of 44 μ/mL lipase was obtained with untreated coconut dregs. The lipase activity was further reduced to only 26 U/mL when *B. stratosphericus* was grown in the basal media without coconut dregs.

### Partial characterisation of *B. stratosphericus* lipase

#### Effects of temperature and pH on lipase activity

**Fig. 3:**
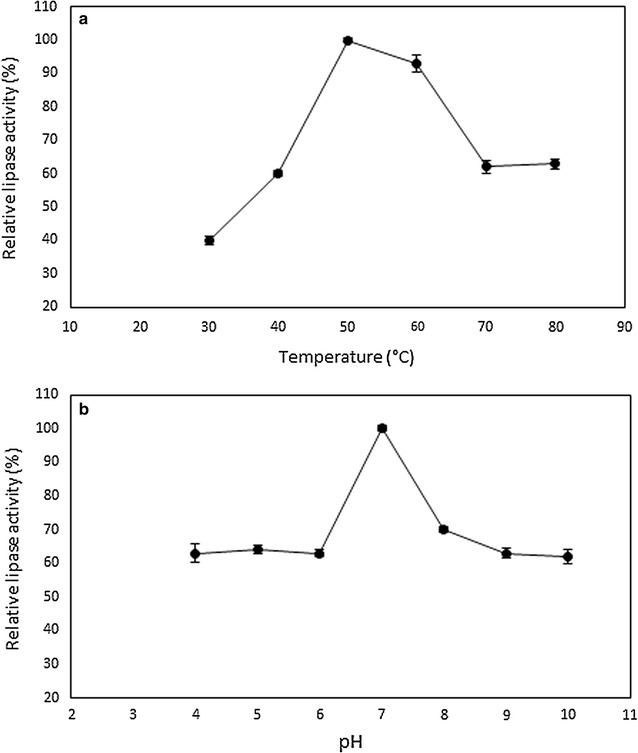
Effect of different temperature and pH on lipase activity. Relative activity of *B. stratosphericus* lipase incubated at different **a** temperature and **b** pH was determined. *Error bars* represent one standard deviation about the mean (n = 3)

As depicted in Fig. 3, the lipase activity has increased with increasing temperature and retained 100 and 93% of its activities at 50 and 60 °C, respectively. Thereafter, the lipase activity dropped to approximately 62% when incubated at 70 and 80 °C. Hence, *B. stratosphericus* lipase has preferred the high temperature of 50 °C for maximal activity although the lipase was active over a broad range of temperature between 30 and 80 °C. The *B. stratosphericus* lipase exhibited its optimum lipolytic activity (100%) at pH 7 and the activity was retained by 60–70% at pH 4–10.

#### Effects of detergent ingredients on lipase stability

**Fig. 4:**
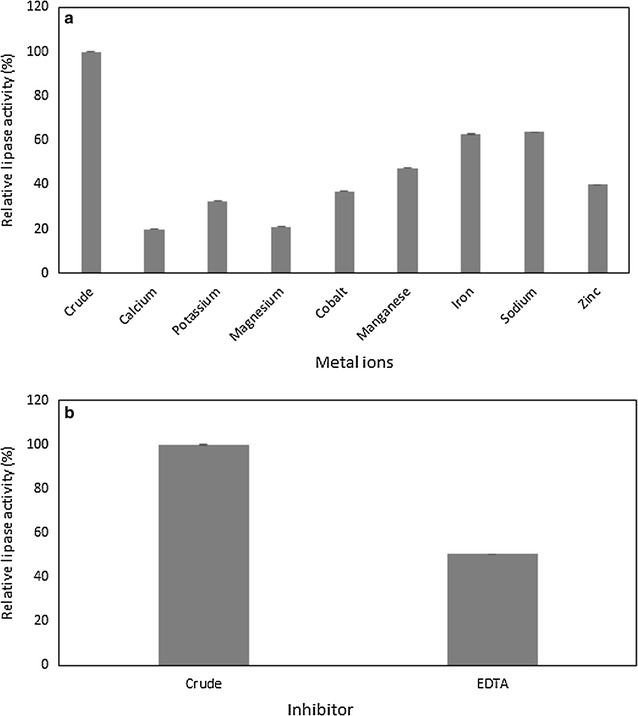
Effect of metal ions and inhibitor on lipase activity. Residual activity of *B. stratosphericus* lipase incubated with 0.01 M of **a** metal ions (Ca^2+^, Co^2+^, Zn^2+^, Na^2+^, K^+^, Fe^3+^, Mn^2+^, and Mg^2+^); and **b** inhibitor [ethylenediaminetetraacetic acid (EDTA)] for 1 h at 50 °C was determined. *Error bar* represents one standard deviation about the mean (n = 3)

The behaviour of *B. stratosphericus* lipase in the presence of a few metal ions is shown in Fig. 4a. The lipase activity was inhibited by all the metal ions (Ca^2+^, Co^2+^, Zn^2+^, Na^2+^, K^+^, Fe^3+^, Mn^2+^, and Mg^2+^) tested at concentration of 0.01 M. Among these, Ca^2+^ and Mg^2+^ have mostly inhibited lipase as can be seen by the residual activity of only 20% after 1 h incubation at 50 °C.

Based on the result in Fig. 4b, lipase was moderately inhibited by 1 mM EDTA and retained 50.49% of its residual activity after 1 h incubation at 50 °C.

**Fig. 5:**
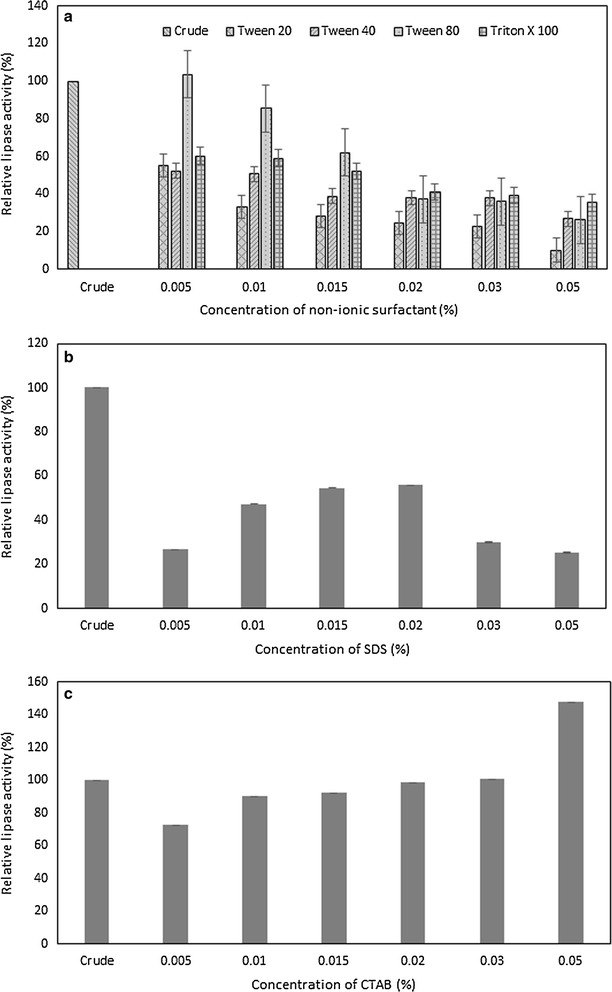
Effect of surfactants on lipase activity. Residual activity of *B. stratosphericus* lipase incubated with various concentrations of **a** non-ionic surfactants (Tween-20, Tween-40, Tween-80 and Triton X-100); **b** anionic surfactant (SDS); and **c** cationic surfactant (CTAB) for 1 h at 50 °C was determined. *Error bar* represents one standard deviation about the mean (n = 3)

The effects of non-ionic surfactants at concentrations in the range of 0.5–5% (v/v) on lipase activity was studied as depicted in Fig. 5a. The result showed that the lipase activity of *B. stratosphericus* has slightly increased by 3.69% from 100% crude residual activity of the control at 0.5% (v/v) of Tween-80. On the other hand, the activity of *B. stratosphericus* lipase was only partially retained when incubated in 0.5% (v/v) of Tween-20, Tween-40 and Triton X-100. In general, all tested non-ionic surfactants showed a similar pattern of decreasing residual activity with increasing concentrations. Among these, the lipase activity was strongly inhibited by 5% (v/v) of Tween-20. Figure 5b shows the effects of anionic surfactant, SDS at concentrations of 0.5–5% (v/v) on *B. stratosphericus* lipase. The highest lipase activity was retained at 55% when incubated in 1.5 and 2% (v/v) of SDS. Meanwhile, lipase activity was enhanced by 47.54% above 100% crude residual activity when incubated with 5% (v/v) of cationic surfactant, CTAB (Fig. 5c).

**Fig. 6:**
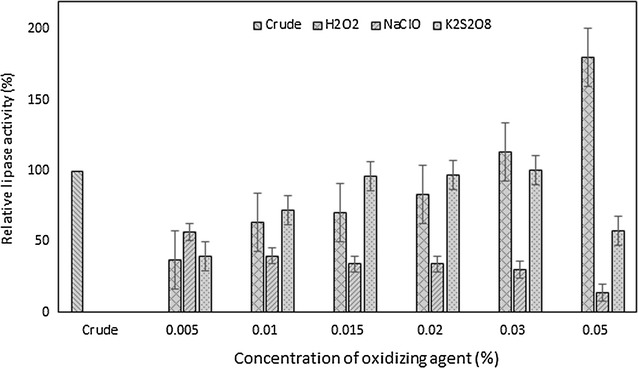
Effect of oxidizing agents on lipase activity. Residual activity of *B. stratosphericus* lipase incubated with various concentrations of H_2_O_2_, NaClO and K_2_S_2_O_8_ for 1 h at 50 °C was determined. *Error bar* represents one standard deviation about the mean (n = 3)

The effects of oxidising agents (H_2_O_2_, NaClO and K_2_S_2_O_8_) were tested on the lipase activity of *B. stratosphericus* as depicted in Fig. 6. It was apparent that the lipase activities were enhanced by the presence of 3 and 5% (v/v) of hydrogen peroxide to 13.05 and 80.30% above 100% crude residual activity, respectively. Lipase was also found stable at 1.5, 2.0 and 3.0% (v/v) potassium persulphate with 96.31, 96.80 and 100.74% relative residual activity, respectively.

#### Effects of commercial detergents on lipase activity

**Fig. 7:**
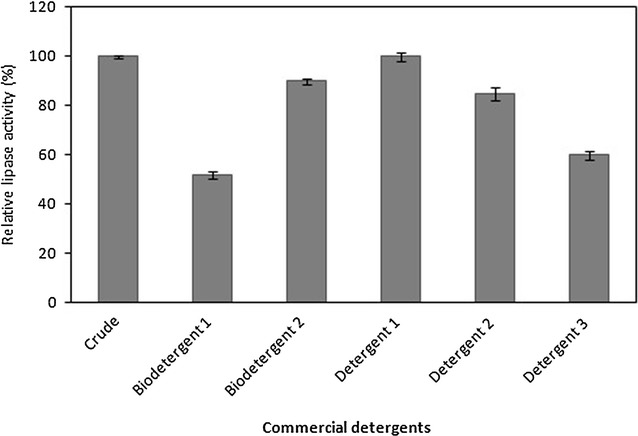
Compatibility of lipase in commercial detergents. Residual activity of *B. stratosphericus* lipase incubated with commercial detergents for 1 h at 50 °C, pH 7 was determined. *Error bar* represents one standard deviation about the mean (n = 3)

Compatibility of crude lipase from *B. stratosphericus* was determined on several commercial detergents. With so many laundry detergents available in local market, only five different brands were selected to be tested. Two of them are biodetergents (biodetergent 1 and biodetergent 2) formulated with unknown enzyme, while the other three are non-biological detergents (detergent 1, detergent 2, detergent 3). Prior the compatibility test, the biodetergents were firstly dissolved into distilled water and heated in a water bath at 90 °C for 1 h to ensure that the availability of endogenous enzymes in the detergents was de-activated. The initial pHs of the diluted detergents were verified and determined to be alkaline. The lipase from *B. stratosphericus* was most stable in detergent 1, detergent 2 and biodetergent 2 with approximately 100, 85 and 90% residual activities, respectively, which were retained after 1 h incubation (Fig. 7). The lowest residual activity was recorded in biodetergent 1 with only 50% residual activity.

## Discussion

Newly isolated *B. stratosphericus* from POME sludge was cultivated in acid pre-treated coconut dregs and characterised for biodetergent compatibility. Although *B. stratosphericus* is believed to primarily live in the stratosphere (Shivaji et al. 2006), atmospheric cycling is thought to bring the stratospheric bacteria to earth, explaining its presence in environments such as POME sludge, deep sea (Odisi et al. 2012) and seawater (Hentati et al. 2016). To date, reports on lipase production by *B. stratosphericus* were seemed very scarce. Among the very few, *B. stratosphericus*—LAMA 585 isolated from South Atlantic deep-sea environment was reported to be a good prospect for producing both lipase and cellulase enzymes (Odisi et al. 2012). Furthermore, the strain was capable of growing well in both rich and minimal medium and producing its mesophilic lipases and cellulases without inducers.

The successful utilisation of lignocellulosic waste including coconut dregs as a carbon source for bacterial growth requires a strain candidate that is also capable to perform cellulose hydrolysis producing fermentable sugars (Maki et al. 2009). To facilitate the enzyme access to the polysaccharides especially cellulose, acid and alkali pre-treatments of the lignocellulosic materials of coconut dregs have been proposed with the intentions to disorganise the plant cell wall structure and to remove lignin. Pre-treatment of lignocelluloses is an essential step to make fermentable sugar available for the microbial biomass (Ashfaque et al. 2014). Nevertheless, incubation time seemed to have an enormous influence on both acid and alkali pre-treatments of coconut dreg. Glucose is the second intermediate product of cellulose biomass and further degradation will lead to the decomposition of 5-hydroxymethyl-furfural (Goto et al. 2004). Hence, an appropriate hydrolysis time is crucial to avoid the degradation of glucose. Bujang et al. (2013) reported 0.38 g/L glucose was hydrolysed from coconut dregs sample only after 30 min incubation with 1% sulfuric acid at 130 °C whilst 48 h was required to obtained 0.2 g/L glucose at 50 °C in this study. These results showed that besides incubation time, incubation temperature also plays a role in the acid hydrolysis rate of coconut dregs.

Carbon source with lipidic content such as coconut dregs may be exploited as an excellent substrate to trigger the associated genes responsible for lipase expression (Thakur et al. 2014). The highest lipase activity (140 U/mL) as observed in the cultivation with acid pre-treated coconuts dregs without the supplementation of coconut oil showed that *B. stratosphericus* prefers the readily available oil in the coconut dregs rather than the supplementary oil as an inducer for lipase production. Furthermore, the ability of *B. stratosphericus* to secrete lipase without supplementary lipid inducer presented the constitutive nature of this strain. In fact, many bacterial lipases were also reported to be constitutive in nature. For instance, lipase produced by *Pseudomonas* sp. BWS-5 was found to be constitutive in nature when there is no increment in lipase production at different olive oil concentrations (Sooch and Kauldhar 2013). Similarly, *Thermomyces lanuginosus* strains have also produced lipase in constitutive nature when different concentrations of olive oil in the medium gave no effect to the lipase production (Sreelatha et al. 2016). The presence of coconut oil (i.e., 3.5%) seemed to have an inhibitory effect on lipase activity. At certain concentrations, the oil layer in the media can interfere with oxygen transfer and may impede cell survival and growth (Kanmani et al. 2015). As expected, untreated coconut dregs did not favour lipase production when only a maximum of 44 U/mL lipase was obtained. Without pre-treatment, lignin in the lignocellulosic material cannot be eliminated. Lignin may block the degradation of cellulose into fermentable sugar by acting as an enzymatic barrier or an inhibitor for cellulase thus inhibit cellulose hydrolysing reaction (Kam et al. 2016). In general, the high yield of lipase obtained in the submerged fermentation using coconut dregs presented in this study has created another value-added for coconut waste.

Preferred detergent lipase should have a sufficient lipase activity in washing solutions and adaptability in broad pH and temperature conditions (Wang et al. 2012). Generally, the pH of washing water is in the alkaline region (Niyonzima and More 2015). The 93–100% relative activity at 50–60 °C and 60–70% relative activity at pH 4–10 indicated the thermostability and broad pH ranges of *B. stratosphericus* lipase. These features have justified its further investigation for biodetergent application.

Metal ions are known to have a significant function in influencing the structure and functions of an enzyme. Metal ions may be required to maintain the stability and activity of lipase enzyme. In this study, *B. stratosphericus* lipase did not depend on the metal ions to stabilise its reaction. This result is contradict with other studies where the lipase activity of *Staphylococcus* sp. ESW had increased by 20% in 2 mM of calcium ion (Cheriff et al. 2011) and calcium ion has also enhanced the lipase activity of *Staphylococcus pasteuri* SNA59 (Aruna and Khan 2014). Hence, a further study is necessary to screen the effects of metal ion concentrations on lipase as it was reported that metal ions at certain concentration will be inhibiting instead of activating the enzyme (Lailaja and Chandrasekaran 2013).

In addition, the effects of inhibitor were studied to understand the involvement of amino acid on the activity of *B. stratosphericus* lipase. Stability in EDTA is essential for a lipase detergent since chelating agent aids in removing ions accounted for water hardness leading to a proper stain removal (Niyonzima and More 2015). However, EDTA is known as a potent inhibitor for metalloenzyme as it can sequester the presence of metal ions (Lailaja and Chandrasekaran 2013). This effect however can generally be overcome by supplementing excess suitable metal ions. *B. stratosphericus* lipase was found to be moderately inhibited by 0.01 M EDTA after 1 h incubation. Nonetheless, metal ions tested in this study were also found to act as inhibitors for *B. stratosphericus* lipase and hence the EDTA inhibitory effects cannot be overcome by treatment with divalent ions at the tested concentrations. Previously, *B. smithii* lipase was also reported to be moderately affected by EDTA at various concentrations (Lailaja and Chandrasekaran 2013).

Besides pH and temperature, a good detergent lipase must be stable in the presence of various detergent ingredients (Niyonzima and More 2015). Surfactants are among the compounds in detergent formulation comprising molecules capable of changing the interfacial characteristics in solutions (Nerurkar et al. 2013). Among the tested non-ionic surfactants (Tween 20, Tween 40, Tween 80, and Triton X-100), only Tween 80 at 0.5% (v/v) has facilitated the enhancement of lipase activity (103.69 U/mL) to above the control residual activity. Similarly, Tween-80 was reported to be a good anolog of lipidic substances and reliable substrate for detergent lipase from *Bacillus sonorensis* 4R (Hemlata et al. 2016). In the meantime, *B. stratosphericus* lipase was strongly inhibited by Tween 20. Some surfactants may cause alterations in the interfacial properties or changes of the lipase active site conformation preventing the binding of lipase to substrate, resulting in enzyme inactivation (Khoramnia et al. 2011). The partial inhibition of the activity of *B. stratosphericus* lipase in SDS might be due to the formation of lipase–anionic surfactant complexes that changes lipase active site conformation and lead to improper enzyme folding as well as causing enzyme inactivation (Prazeres et al. 2006) or obstructing the site for substrate adhesion (Khoo and Ibrahim 2009). Previously, several detergent compatible lipases were also reported to be inhibited by SDS. For instant, lipase from *Pseudomonas aeruginosa* KM110 had loss 30% of its activity (Mobarak-Qamsari et al. 2011) while lipase from *Acinetobacter calcoaceticus* loss 76.3% activity compared to control when incubated in 1% SDS (Wang et al. 2012). In contrast, CTAB has strongly helped to boost the activity of *B. stratosphericus* lipase. This increment can be attributed to the ability of CTAB as a cationic surfactant to solubilise the lipolysis products to prevent their interfacial accumulation (Jurado et al. 2007).

Oxidising or bleaching agents are frequently added in the detergent formulation for digestion and decolourisation of stains and organic materials during washing. Good stability of enzyme in bleach is relatively uncommon and can only be achieved by site-directed mutagenesis or protein engineering (Dutta and Ray 2009). Surprisingly, the present lipase was inherently stable towards some oxidising agents particularly hydrogen peroxide at high concentrations [3–5% (v/v)]. Bisht et al. (2013) also reported that the activity of alkaline lipase from *Pseudomonas aeruginosa* mutant was enhanced in the presence of hydrogen peroxide with residual activity of 104.5%. Meanwhile, a few other lipases only managed to either retained their residual activity equivalent to control (100%) (Costa-Silva et al. 2014; Chauhan et al. 2013; Dutta and Ray 2009) or moderately (Chauhan et al. 2013; Wang et al. 2012; Weerasooriya and Kumarasinghe 2012) to strongly (Khoo and Ibrahim 2009) inhibited by hydrogen peroxide. The stability profile of *B. stratosphericus* lipase in oxidising agents proved its potential in detergent formulation.

The good compatibility of *B. stratosphericus* lipase with both biological and non-biological detergents may be mostly attributed to its better stability performance in alkaline pH. The ability of the enzyme to retain above 80% activity in some selected detergents showed that although they are not strictly belonging to the alkaline lipase group, the enzyme can effectively serve as an additive in biodetergents with the right formulation. Furthermore, the thermostability feature of *B. stratosphericus* lipase is an added value as high temperatures are often preferable to remove difficult stain in cotton and synthetic materials (Bora and Bora 2012). Thus, it can be stated that the lipase from *B. stratosphericus* is suitable to be used as an additive in detergent formulation.

## Abbreviations

ADF: acid detergent fiber
NDF: neutral detergent fiber
ADL: acid detergent lignin
POME: palm oil mill effluent
LB: Luria–Bertani
CMC: carboxymethyl cellulose
pNPL: *p*-nitrophenyl-laurate
pNP: *p*-nitrophenol
SDS: sodium dodecyl sulphate
EDTA: ethylenediaminetetra acetic acid
CTAB: cetyltrimethylammonium bromide
